# Comparative Evaluation of the Effect of Microwave, 1% Sodium Hypochlorite, and Sodium Perborate Disinfection on the Color Stability of Two Nanoparticle-Reinforced Heat-Polymerized PMMA Denture Base Resins: An In Vitro Study

**DOI:** 10.7759/cureus.67350

**Published:** 2024-08-20

**Authors:** Mrugnayani P Patel, Vishal B Parmar, Diptesh S Rami, Vishal Rakesh Trivedi, Dhaara M Rana, Dhara N Bajania

**Affiliations:** 1 Department of Prosthodontics and Crown & Bridge, Manubhai Patel Dental College, Vadodara, IND; 2 Department of Prosthodontics and Crown & Bridge, Narsinhbhai Patel Dental College and Hospital, Sankalchand Patel University, Visnagar, IND; 3 Department of Prosthodontics and Crown &amp; Bridge, Ahmedabad Municipal Corporation (AMC) Dental College and Hospital, Ahmedabad, IND; 4 Department of Prosthodontics and Crown &amp; Bridge, Government Dental College and Hospital, Jamnagar, IND

**Keywords:** sodium hypochlorite, microwave irradiation, sodium perborate, disinfection methods, color stability, spectrophotometer, nanoparticle reinforcement, zirconium oxide, titanium dioxide, pmma

## Abstract

Introduction

Older adults experience significant improvement in their quality of life by using removable prosthetics to replace missing teeth. Poly(methyl methacrylate) (PMMA) has become the most popular material for denture bases due to its ease of use and affordability. Recently, scientists have started adding nanoparticles like titanium dioxide (TiO2) and zirconium oxide (ZrO2) to PMMA to enhance its physical properties. These resins with nanoparticles need to stay the same color after being disinfected in different ways if they are going to be used for a long time. So, the purpose of this investigation was to assess whether or not there exists any difference between two kinds of thermally cured acrylic resin for artificial tooth bases strengthened with nanoparticles when subjected to various chemical sterilizers alongside microwave irradiation, as well as determine their comparative colorfastness levels.

Materials and methods

In this lab experiment, we tested how well 5% TiO2 and 7% ZrO2 nanoparticle-reinforced PMMA resins held their color when exposed to microwave irradiation, 1% sodium hypochlorite, or sodium perborate disinfection. We made 120 specimens shaped like discs; half were treated using one method, while the other half were treated using a different method. Color was measured at baseline (T0), after one cycle (T1), after five cycles (T2), and after six months (T3) using a reflectance spectrophotometer, which calculates the color difference (∆E).

Results

All three methods of disinfection caused significant color changes (p<0.001); however, sodium perborate caused the least amount of change, followed by 1% sodium hypochlorite and microwave irradiation. The mean ∆E values showed that after one day, there was a change in color by 1.1 due to microwave disinfection, which increased to 5.7 after five days; on the other hand, for 1% sodium hypochlorite, the change was recorded as 0.7 after one month and 1.6 after three months and finally reached up to 2.6 after six months, while sodium perborate showed the least amount of change, with ∆E values recorded as 0.2 after one month, 0.5 after three months, and 0.8 after six months.

Conclusion

Sodium perborate proved to be the most effective disinfectant for maintaining color stability in 5% TiO2 and 7% ZrO2 nanoparticle-reinforced PMMA resins, thus making it ideal for routine disinfection. Therefore, according to this study, sodium perborate should be used as a disinfection method because it results in minimal color change in nanoparticle-reinforced PMMA dentures.

## Introduction

Throughout history, different substances such as metal alloys, ivory, and wood have been used to make dentures, but since 1937, poly(methyl methacrylate) (PMMA) has been introduced into dentistry as a new material for making denture bases because of its easy handling characteristics combined with low costs [1-3].

Since the mid-20th century, PMMA acrylic resin has been the preferred material for fabricating full or partial removable dental appliances. This is due to its numerous advantages, such as its good biocompatibility with oral tissues, excellent aesthetic results, desirable physical and chemical properties, including resistance against water absorption, and easy manipulation during processing stages at affordable prices [4]. Regardless of what kind of material is used, patients should always ensure that they clean their prostheses frequently through physical methods that involve brushing with soft, non-abrasive bristles [5].

Nanotechnology, which is an area dealing with control over matter at the atomic or molecular scale, has been utilized so far mainly towards enhancing mechanical features displayed by denture base materials; examples include PMMA chemical modification alternatives, fiber reinforcement development, and macro- and nano-filler utilization, among others [6]. It has been proven that the incorporation of nanometer-sized particles as reinforcement agents can improve the physical properties of resins made from acrylics [6]. More recent studies have concentrated on incorporating inorganic nanoparticles within PMMA, aiming at improving its performance characteristics such as shape selectivity, size sensitivity, concentration dependence, and polymer matrix interaction effect on the resultant properties of polymer nanocomposite mixtures [7].

Zirconium oxide (ZrO2, zirconia) particles are most commonly used for mechanically strengthening polymers through reinforcement effects in the case of reinforced PMMA composites. For this methodology to be successful, there should be good bonding between the resin phase and the dispersed particles, thus leading to homogeneous distribution throughout the composite material and thereby enhancing the flexural strength properties observed in such systems. Nano-sized ZrO2 powders have been preferred over other metal oxide nanoparticles because they are biocompatible and have excellent aesthetic qualities due to their white coloration [8]. Furthermore, ZrO2 is characterized by high toughness values combined with superior mechanical strength levels as well as good wear resistance ability, which makes it a suitable candidate filler material when trying to improve upon the mechanical behavior exhibited by acrylic resins [9].

Titanium dioxide (TiO2) as nanoparticles was used in previous studies since it increased the surface hydrophobicity, reduced the adherence of biomolecules, aided in coloring, had antimicrobial properties, and improved the mechanical properties of PMMA resins. Tests that had been conducted previously with TiO2 incorporated in PMMA showed increased mechanical properties with extended strong interfacial adhesion [3]. The addition of 5% TiO2 to PMMA leads to an increase in flexural strength, attributed to the reduced filler size, which contributes to improved fracture resistance of the material.

Another method that has been shown to effectively disinfect dentures is microwave irradiation. This process is simple, quick, and inexpensive and does not cause any discoloration or odor changes in the prostheses; however, appliances containing metals cannot be disinfected using microwaves. Although simple and fast, the short- and long-term effects of microwave treatment on materials used in denture fabrication procedures remain unknown since no standard protocols have been established yet for microwave oven therapy [10,11]. Disinfection using a microwave does not require special storage or an expiration date, therefore making it one of the best methods for sterilizing against *Candida albicans* infections [12].

Since the oral cavity is made up of a microbial environment, it is necessary to keep dentures clean. PMMA-based resins can change color when exposed to disinfecting processes and oral fluids over time [2,13,14]. Some commonly used disinfectants are sodium hypochlorite (1%), sodium perborate, and microwave sterilization. These agents have been found to affect the color stability properties of traditional denture base resin materials, as reported in numerous studies. However, there is limited information about how these agents affect nanoparticle-reinforced PMMA resins [5].

According to Uludamar et al.'s in vivo study, different durations of treatment with sodium perborate tablets showed 60 minutes to be the most effective duration against *Candida* species [15]. This hypothesis suggests that incorporating nanoparticles into thermally cured acrylic resins will improve their durability and resistance to color changes when exposed to different disinfection methods, including chemical sterilizers and microwave irradiation. The purpose of this investigation was to assess whether or not there exists any difference between two kinds of thermally cured acrylic resin for artificial tooth bases strengthened with nanoparticles when subjected to various chemical sterilizers alongside microwave irradiation, as well as determine their comparative colorfastness levels. This evaluation will contribute towards creating methods for disinfecting complete dentures so that they do not lose their inherent qualities but at the same time promote oral cleanliness.

## Materials and methods

This lab test was conducted in the Department of Prosthodontics and Crown & Bridge of K.M. Shah Dental College and Hospital, Piparia, Vadodara, after obtaining approval from the institutional ethical committee (approval number: SVIEC/ON/DENT/BNPG16/D17005). A proforma sheet recorded basic information about samples and color stability. According to a study by Andreotti et al., a one-way analysis of variance (ANOVA) showed that at least eight samples per group were required to detect a mean color change of 0.26 with a standard deviation of 0.13 at a 5% risk level and an 80% power [1].

As three time intervals were planned, a minimum total of 12 samples per group was necessary for this research. Three disinfection subgroups, microwave, 1% sodium hypochlorite, and sodium perborate, were included in this study. Five samples per time interval were taken to achieve sufficient power, which made it a total of 120 samples (60 for each nanoparticle group; 5% TiO2 and 7% ZrO2). We used modeling wax, dental stone, separating medium, aluminum foil, petroleum jelly, heat-cured denture base resin universal polishing paste, and M% TiO2 and M% ZrO2 nanoparticles as materials for specimen preparation. Group A and Group B both have 60 samples each. Each group is divided into three subgroups. Each subgroup has 20 samples each. All the same 20 samples had undergone the time interval. T0, T1, T2, and T3 have the same 20 samples. The instruments used for specimen preparation included a metallic die, a Bard-Parker (BP) knife handle and blade, and a rubber bowl spatula. We used a mechanical vibrator, a micromotor for denture finishing and polishing, a paintbrush, a porcelain mixing jar, a dental flask clamp, glass slabs, and airtight plastic containers as instruments for specimen preparation. 

**Figure 1:**
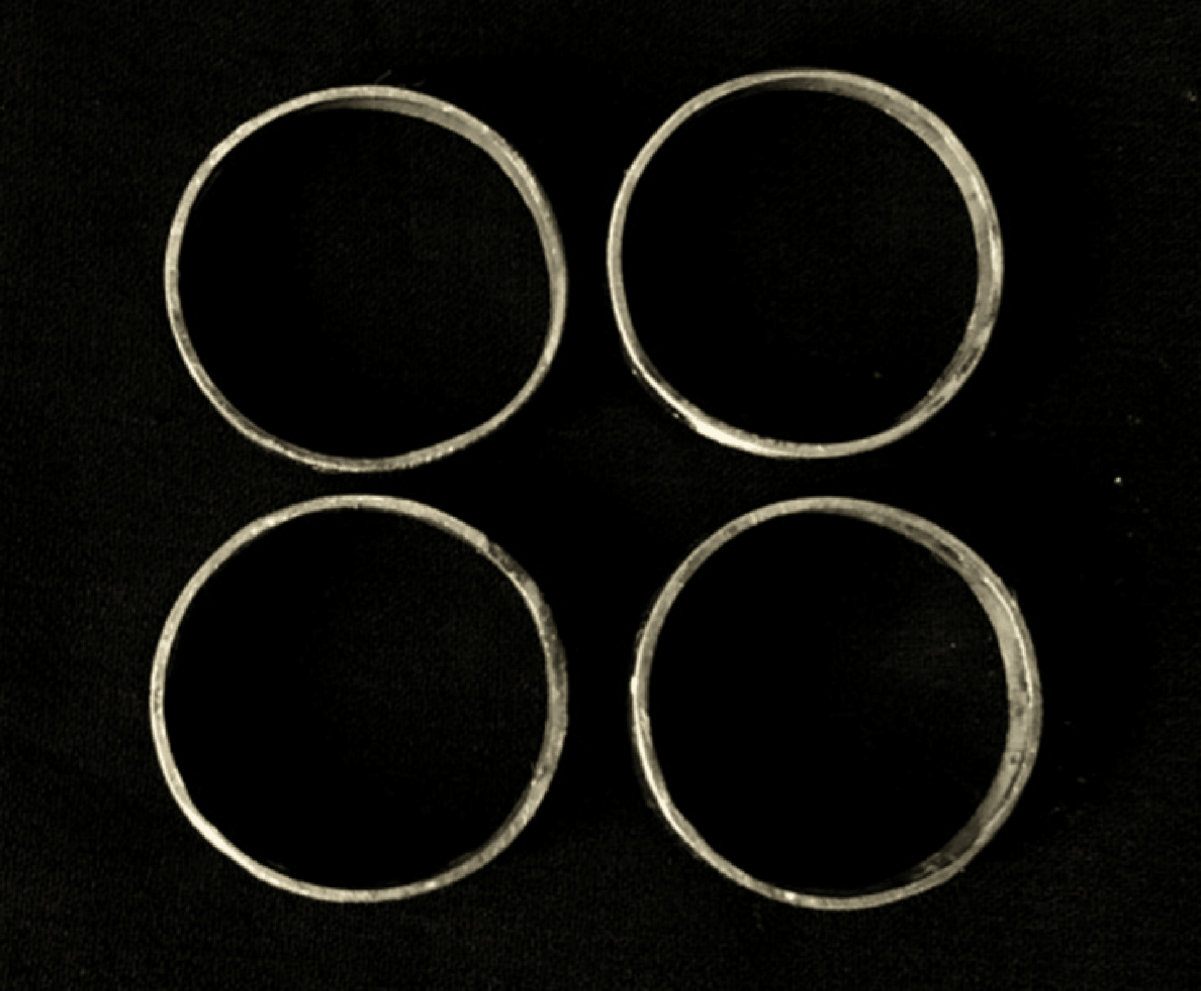
Metallic die (disc shape with a diameter of 3 cm (30 mm) and a thickness of 0.3 cm (3 mm))

We used equipment such as a ball milling apparatus, an acrylic polishing lathe, and an incubator. In specimen preparation, disc-shaped samples with a diameter of 30 mm and a thickness of 3 mm were created using a metallic die, which was invested in conventional denture flasks in type II dental stone [2]. The specimens were kept for 12 hours for bench curing to ensure less denture base discrepancy, as recommended by Consani et al. [2]. For microwave disinfection cycles, samples were stored in a glass beaker. The microwave oven was set at 650 wattage (W) for three minutes referred to as one disinfection cycle of microwave irradiation. Group samples were disinfected one after the other, according to the number of cycles of microwave disinfection. Thus, for one microwave disinfection, the microwave oven was set at 650 W for three minutes. This one cycle was repeated five times for five microwave disinfection without any breaks. After the completion of microwave disinfection, all the samples were subjected to color stability (Figure 1).

**Figure 2:**
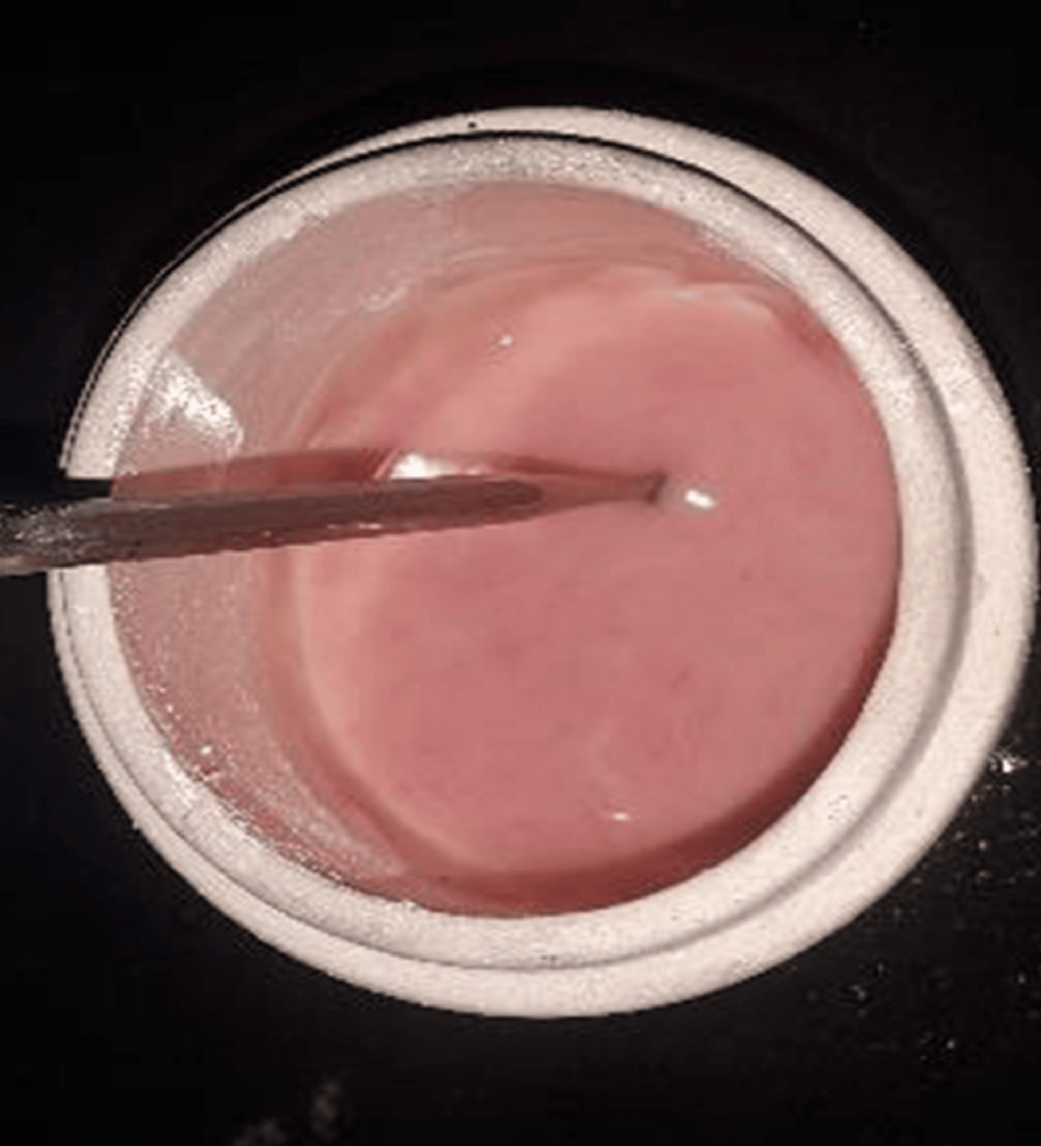
Mixing of nanoparticle-reinforced PMMA PMMA: poly(methyl methacrylate)

The powder component of heat-cured PMMA was modified by the incorporation of 5% TiO2 and 7% ZrO2 nano-filler powders procured from Reinste Nano Ventures Pvt. Ltd., New Delhi, India. The specimens were fabricated using a metallic die, measuring 3 cm in diameter and 0.3 cm in thickness. The nanoparticles were individually weighed with an electronic balance, accurate to four decimal places in grams. We mixed the nanoparticles with the polymer of heat-polymerized acrylic resin using a ball milling apparatus at 60 revolutions per minute (rpm) for approximately four hours to ensure homogeneous mixing [5,6]. Then, nanoparticle-reinforced PMMA was mixed with monomer as per the manufacturer's instructions. Modified PMMA was packed into the moulds to form 120 samples, which were then polished using a universal polishing paste composed of aluminum oxide on a soft cloth wheel for one minute at a speed of 3000 rpm [7] (Figure 2).

**Figure 3:**
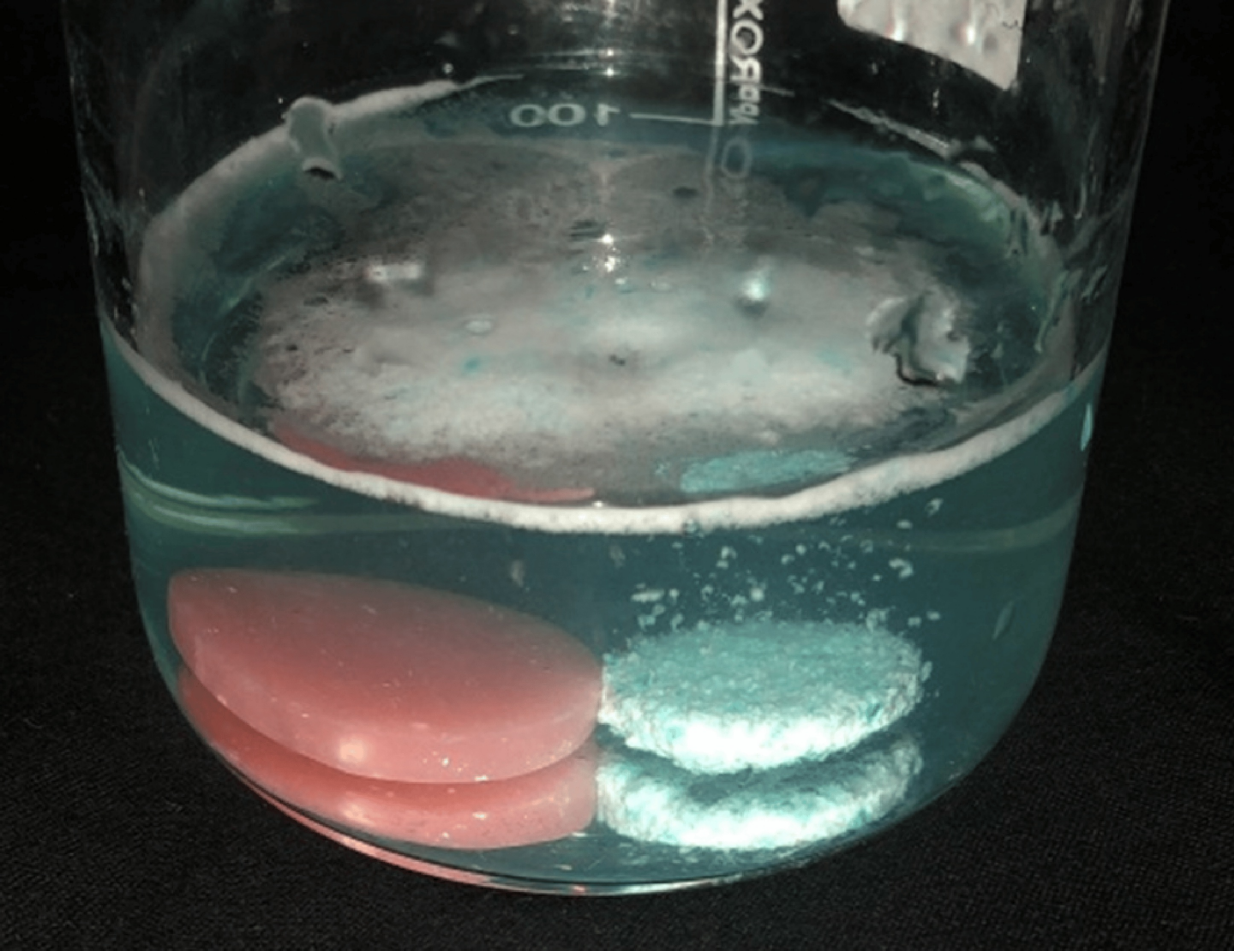
Specimen in Fittydent tablets (sodium perborate)

After polishing, all specimens were stored in normal water at 37 degrees Celsius for 24 hours in an incubator. The specimens were divided into two main groups, each containing 60 samples, and further divided into six subgroups, each containing 20 samples based on the disinfecting agent, i.e., microwave, 1% sodium hypochlorite, and sodium perborate. Each subgroup was further divided into three different time intervals: T0 (baseline color), T1 (after one cycle/one month), T2 (after five cycles/three months), and T3 (after six months) [8,9]. To disinfect, Fittydent tablets were used by immersing the specimens every day for one hour and then in 1% sodium hypochlorite for 10 minutes. After that, we washed the specimens with water and placed them in distilled water until the next soaking trial, continuing to immerse them regularly for the next six months. For microwave disinfection, 150 ml of water was used to subject specimens to 650 W within three minutes, where cycles were observed on the first and fifth days (Figure 3).

The reflectance spectrophotometer was utilized to observe color changes in denture base materials (SpectraScan 5100, Premier Colorscan Instruments, Navi Mumbai, India). The difference between colors (∆E) was calculated through the Commission Internationale de l'Eclairage (CIE) Lab* system; however, the ∆E value is also presented in the National Bureau of Standards (NBS) units so that it can be clinically related as well. In terms of statistical analysis, independent t-tests, repeated measures ANOVAs, paired t-tests, and one-way ANOVAs followed by post hoc tests have been conducted to compare color stability among different sample groups.

## Results

Table 1 shows the initial color values of the specimens mixed with 5% TiO2 and 7% ZrO2 nanoparticles that were exposed to different disinfection techniques.

**Table 1:** Baseline (T0) color values Lightness (L*), redness or greenness (a*), and yellowness or blueness (b*)

Specimen no.	Subgroup 1: microwave	Subgroup 2: 1% sodium hypochlorite	Subgroup 3: sodium perborate
	L*	a*	b*
1	44.177	21.537	1.585
2	44.217	21.074	1.823
3	43.259	19.645	1.561
4	44.111	21.404	1.688
5	43.724	20.358	1.747

For each sample, lightness (L*), redness or greenness (a*), and yellowness or blueness (b*) were measured at T0. The L* values ranged from 43.259 to 44.217, indicating small variations in brightness among the samples. Meanwhile, a* varied between 19.645 and 21.537, representing the red-green coordinate, whereas b* ranged from 1.561 to 1.823, denoting the yellow-blue coordinate. These baseline figures serve as benchmarks against which subsequent color changes caused by disinfection can be evaluated.

Table 2 displays the color alteration for the microwave subgroup from day 1 to day 5 through the E measurement, demonstrating a gradual increase over time.

**Table 2:** Color change (ΔE) for the microwave subgroup

Specimen no.	Microwave (after one day)	Microwave (after five days)
1	0.7	6.0
2	1.8	5.8
3	1.2	5.4
4	1.5	5.5
5	1.1	5.5

On the first day, the values ranged between 1.7 and 1.8, with a mean value of approximately 1, while on the fifth day, the ranges significantly increased, ranging from 5.4 to 6, with a mean value of approximately 5.7. This indicates that the specimens used the microwaving method for disinfection over this five-day period.

Color change (ΔE) among the sodium hypochlorite subgroup, where ΔE gradually increases over time, implies a progressive change in shade. Starting at month 1, there are slight differences between various shades, but by month 3, these differences have become more pronounced, as seen through a range of 0.7-1.0, and after six months, the range has increased further from 1.1 to 1.4. This shows the cumulative effect of prolonged exposure to sodium hypochlorite, which leads to cumulating changes in colors observed within specimens (Table 3).

**Table 3:** Color change (ΔE) for the 1% sodium hypochlorite subgroup

Specimen no.	Sodium hypochlorite (after one month)	Sodium hypochlorite (after three months)	Sodium hypochlorite (after six months)
1	0.5	0.7	1.1
2	0.7	0.8	1.2
3	0.9	0.9	1.3
4	1.0	1.0	1.4
5	0.8	0.9	1.3

Table 4 shows mean ∆E values for subgroups exposed to different disinfection techniques at various time intervals.

**Table 4:** Mean value of color change (ΔE) for subgroups at various time intervals

Groups	Time intervals	Mean value of color change (ΔE)
Subgroup 1 (microwave)	After one day	1.1
	After five days	5.7
Subgroup 2 (1% sodium hypochlorite)	After one month	0.7
	After three months	1.6
	After six months	2.6
Subgroup 3 (sodium perborate)	After one month	0.2
	After three months	0.5
	After six months	0.8

The average color change was calculated among subgroups using designated formulae and recorded in this table against each subgroup label together with timing details; microwave showed the highest mean delta E value, being equal to 1.1 during the first day, whereas five days later, microwave had the greatest increase in mean delta E value, which is equal to 5.7. On the other hand, 1% sodium hypochlorite revealed steadily increasing means over months, with its lowest measurement being 0.7 after one month, then 1.6 after three months, and 2.6 after six months, representing higher levels. Compared to each time frame, the change rates were significantly lower than those observed between corresponding periods of time when looking at microwave data alone (the microwave group deteriorated after five cycles, and the least color change (∆E) was found in sodium perborate disinfection of 5% TiO2 and 7% ZrO2 nanoparticle-reinforced PMMA specimens when compared with 1% sodium hypochlorite disinfection and microwave irradiation). Thus, it can be concluded that 5% TiO2 and 7% ZrO2 nanoparticle-reinforced PMMA specimens can be better disinfected in sodium perborate (Fittydent tablets) followed by microwave, sodium perborate, and so on. There was statistical significance, particularly in the comparison between the subgroups that were subjected to the sodium perborate and 1% sodium hypochlorite methods.

## Discussion

Acrylic resins represent most false teeth worldwide due to their low cost and ease of use. However, there are some downsides to this type of material, such as the fact that it can stain easily and become rough on the surface when exposed to common disinfectants. When it comes to denture-based acrylic resin, keeping up appearances is important because color stability matters too. Causes of color change include the accumulation of stains, the absorption of water, the degradation of constituents, the dissolution of intrinsic dyes, and roughness at surfaces, among others. Brightly colored foods and drinks, such as those found in denture bases, can significantly alter the shade of dentures. Additionally, immersion cleansers can also have an impact on these polymers. It's recommended that older people who find cleaning harder should clean their dentures more frequently using both chemical solutions and mechanical methods [12]. Oxygen-releasing agents are one type, while hypochlorite solutions fall under another category called immersion cleansers, where effervescent tablets containing alkaline peroxide are used for chemical and mechanical cleaning actions through oxygen bubbles released into the water.

Mostly, a disinfection method should effectively kill germs without harming the denture base material itself. Everyday use of these cleansers prevents microbes from colonizing our mouths through false teeth, but it also affects physical and mechanical properties like hardness or color [13]. These properties can vary depending on what happens during the mixture process between PMMA and other chemicals, such as plasticizers. However, compatibility between these chemicals plays a crucial role, ensuring that none negatively interfere with one another. For example, surface roughness should not interfere with hardness values. PMMA is commonly used in prosthodontics because it has better aesthetics compared to other materials, is easy to handle, is non-toxic, and is cheap.

If the optical appearance is good, then all will be well since we want people who wear false teeth to not have their self-esteem damaged further due to bad-looking ones caused by poor-quality materials used when making them or during repair work after they break down over time [8]. Many efforts have been made to improve properties such as strength, but these efforts have failed so far because certain base components may undergo chemical changes that affect overall performance. However, adding nanoparticles improves physical attributes, thereby increasing flexural strength up to a point where excessive addition adversely affects this property. Although research has been quite extensive in terms of discovering ways through which we could improve the physical properties associated with denture bases, more should be done, especially when it comes to finding out what happens if any part gets infected or contaminated by various disinfectants used alongside cleansing solutions that contain nanoparticles. It is therefore important for us to know about the impacts different methods of disinfection will have on colored, heat-cured nanoparticle-filled PMMA resins for dentures [7].

The main concern here pertains to long-term disinfections, as they aid in reducing the risk of infections. However, when contaminated base plates are worn for extended periods without proper care, they serve as breeding grounds for bacteria that could later infiltrate the wearer's body, leading to serious illnesses and even fatal situations. Microwaves emit electromagnetic energy that produces effects different from those of conventional heating. The precise mechanism by which this energy destroys microorganisms remains unclear, whether through thermal means or non-thermal processes [6,7]. Chemical procedures, although effective, may take too long to be practical for chairside use. In contrast, microwave irradiation offers a low-cost, quick, and chemical-free method for sterilizing infected areas within resinous materials, such as denture bases contaminated with *Candida* and other fungi [9]. While some studies found no significant changes in the dimensions of dentures subjected to microwaves, others indicated greater efficacy over sodium hypochlorite solution. In these experiments, more water was used, as moist conditions are believed to encourage microbial growth, making them more infectious than those that dry out quickly.

The daily use of denture cleansers can influence the properties of denture base materials. There are a variety of chemical denture cleansers, such as 5.25% sodium hypochlorite solutions, that kill microorganisms effectively but may erode some substances at higher concentrations [15]. Low concentrations (0.5%) of hypochlorite solutions are also effective. In this research, 1% sodium hypochlorite and sodium perborate were used for immersion procedures. Sharma et al. [16] have concluded that immersion in a 1% sodium hypochlorite solution for three months caused an influence on the surface roughness and flexural strength of heat-cured denture base resin, suggesting caution for long-term use while recommending Fittydent and 100% vinegar as routine chemical cleansers for long-term use. 

This research merely looked at how stable colors were over time, a drawback because it ignores other factors too. Future studies could, for instance, measure surface roughness and dimensional changes such as hardness or distortion while also taking into account longer durations and more temperature changes caused by thermocycling with different types of food coloring agents mixed into various beverages. These factors could potentially affect the stability of reinforced resins differently when tested against each other under similar conditions. Moreover, given the limitations of this study, which only examined objects outside of living organisms, researchers could conduct another experiment using animals. However, this experiment would not take into account the conditions inside people's mouths, making it impossible to determine whether the findings would apply to patients. Sodium perborate caused the least amount of discoloration among 7% ZrO2 and 5% TiO2 nanoparticle-strengthened PMMA samples over a six-month period, followed by 1% sodium hypochlorite and microwave irradiation. Since the research conducted is in vitro, it eliminates the factual data that may differ when the patient's oral environment comes into play. The presence of saliva, different temperatures, the normal microflora of the oral cavity, and the forces of the oral musculature may vary the results than those derived from this study.

The strengths of this in vitro study emphasize its novelty in addressing the color stability of nanoparticle-reinforced PMMA resins subjected to various disinfectants, a significant gap in the existing literature. The study's rigorous methodology, involving multiple time intervals, a substantial number of specimens, and the use of a validated spectrophotometer, adds to its reliability. Moreover, the application of appropriate statistical analyses enhances the scientific rigor of the findings. However, it is essential to acknowledge the limitations, including the in vitro setting that may not replicate the complex oral environment, the relatively short six-month study duration, and the limited scope focusing solely on color stability. Future research should aim to conduct long-term in vivo studies and explore additional properties such as surface roughness, hardness, and dimensional changes to build on these foundational findings.

This study's limitations highlight the challenges in translating laboratory findings to clinical applications. Conducted in an in vitro setting, it does not fully replicate the complex and dynamic conditions of the human oral cavity, where factors like saliva, temperature fluctuations, and the presence of oral microflora can significantly impact the performance and longevity of denture materials. The six-month duration, while providing valuable insights into short-term effects, may not accurately predict long-term outcomes. Additionally, focusing solely on color stability without assessing other critical properties such as surface roughness, hardness, and dimensional changes may limit the applicability of the findings to real-world clinical scenarios. Future research should prioritize long-term in vivo studies to better understand how nanoparticle-reinforced PMMA resins behave in the diverse oral environments of patients, ensuring more comprehensive evaluations that can inform clinical practice effectively.

## Conclusions

The in vitro study demonstrated that among the tested disinfection methods, sodium perborate resulted in the least color change in 5% TiO2 and 7% ZrO2 nanoparticle-reinforced PMMA specimens compared to 1% sodium hypochlorite and microwave irradiation. Statistical analysis confirmed significant differences in color stability, with sodium perborate proving the most effective. This finding underscores sodium perborate as the preferred choice for maintaining the color stability of nanoparticle-reinforced PMMA dentures. Therefore, it is recommended to disinfect 5% TiO2 and 7% ZrO2 nanoparticle-reinforced PMMA dentures using sodium perborate to minimize color alteration, ensuring prolonged aesthetic appeal and durability.
